# *Trypanosoma cruzi* surface mucins are involved in the attachment to the *Triatoma infestans* rectal ampoule

**DOI:** 10.1371/journal.pntd.0007418

**Published:** 2019-05-20

**Authors:** María de los Milagros Cámara, Virginia Balouz, Camila Centeno Cameán, Carmen R. Cori, Gustavo A. Kashiwagi, Santiago A. Gil, Natalia Paula Macchiaverna, Marta Victoria Cardinal, Francisco Guaimas, Maite Mabel Lobo, Rosa M. de Lederkremer, Carola Gallo-Rodriguez, Carlos A. Buscaglia

**Affiliations:** 1 Instituto de Investigaciones Biotecnológicas-Instituto Tecnológico de Chascomús (IIB-INTECh), Universidad Nacional de San Martín (UNSAM) and Consejo Nacional de investigaciones científicas y técnicas (CONICET), Buenos Aires, Argentina; 2 Universidad de Buenos Aires, Facultad de Ciencias Exactas y Naturales, Departamento de Química Orgánica, Pabellón 2, Ciudad Universitaria, C1428EGA Buenos Aires, Argentina; 3 CONICET-UBA, Centro de Investigación en Hidratos de Carbono (CIHIDECAR), C1428EGA Buenos Aires, Argentina; 4 Laboratorio de Eco-Epidemiología, Facultad de Ciencias Exactas y Naturales, Universidad de Buenos Aires e Instituto de Ecología, Genética y Evolución de Buenos Aires (IEGEBA), UBA-CONICET, C1428EGA Buenos Aires, Argentina; Harvard School of Public Health, UNITED STATES

## Abstract

**Background:**

*Trypanosoma cruzi*, the agent of Chagas disease, is a protozoan parasite transmitted to humans by blood-sucking triatomine vectors. However, and despite its utmost biological and epidemiological relevance, *T*. *cruzi* development inside the digestive tract of the insect remains a poorly understood process.

**Methods/Principle findings:**

Here we showed that Gp35/50 kDa mucins, the major surface glycoproteins from *T*. *cruzi* insect-dwelling forms, are involved in parasite attachment to the internal cuticle of the triatomine rectal ampoule, a critical step leading to its differentiation into mammal-infective forms. Experimental evidence supporting this conclusion could be summarized as follows: i) native and recombinant Gp35/50 kDa mucins directly interacted with hindgut tissues from *Triatoma infestans*, as assessed by indirect immunofluorescence assays; ii) transgenic epimastigotes over-expressing Gp35/50 kDa mucins on their surface coat exhibited improved attachment rates (~2–3 fold) to such tissues as compared to appropriate transgenic controls and/or wild-type counterparts; and iii) certain chemically synthesized compounds derived from Gp35/50 kDa mucins were able to specifically interfere with epimastigote attachment to the inner lining of *T*. *infestans* rectal ampoules in *ex vivo* binding assays, most likely by competing with or directly blocking insect receptor(s). A solvent-exposed peptide (smugS peptide) from the Gp35/50 kDa mucins protein scaffolds and a branched, Gal*f*-containing trisaccharide (Gal*f*β1–4[Gal*p*β1–6]GlcNAcα) from their *O*-linked glycans were identified as main adhesion determinants for these molecules. Interestingly, exogenous addition of a synthetic Gal*f*β1–4[Gal*p*β1–6]GlcNAcα derivative or of oligosaccharides containing this structure impaired the attachment of Dm28c but not of CL Brener epimastigotes to triatomine hindgut tissues; which correlates with the presence of Gal*f* residues on the Gp35/50 kDa mucins’ *O*-glycans on the former but not the latter parasite clone.

**Conclusion/Significance:**

These results provide novel insights into the mechanisms underlying *T*. *cruzi-*triatomine interplay, and indicate that inter-strain variations in the *O*-glycosylation of Gp35/50 kDa mucins may lead to differences in parasite differentiation and hence, in parasite transmissibility to the mammalian host. Most importantly, our findings point to Gp35/50 kDa mucins and/or the Gal*f* biosynthetic pathway, which is absent in mammals and insects, as appealing targets for the development of *T*. *cruzi* transmission-blocking strategies.

## Author summary

Chagas disease, caused by the protozoan *Trypanosoma cruzi*, is a life-long and debilitating neglected illness of major significance to Latin America public health, for which no vaccine or adequate drugs are yet available. In this scenario, identification of novel drug targets and/or strategies aimed at controlling parasite transmission are urgently needed. By using *ex vivo* binding assays together with different biochemical and genetic approaches, we herein show that Gp35/50 kDa mucins, the major *T*. *cruzi* epimastigote surface glycoproteins, specifically adhere to the internal cuticle of the rectal ampoule of the triatomine vector, a critical step leading to their differentiation into mammal-infective metacyclic forms. *Ex vivo* binding assays in the presence of chemically synthesized analogs allowed the identification of a solvent-exposed peptide and a branched, galactofuranose (Gal*f*)-containing trisaccharide (Gal*f*β1–4[Gal*p*β1–6]GlcNAcα) as major Gp35/50 kDa mucins adhesion determinants. Overall, these results provide novel insights into the mechanisms underlying the complex *T*. *cruzi-*triatomine interplay. In addition, and since the presence of Gal*f*-based glycotopes on the *O*-glycans of Gp35/50 kDa mucins is restricted to certain parasite strains/clones, they also indicate that the Gal*f*β1–4[Gal*p*β1–6]GlcNAcα motif may contribute to the well-established phenotypic variability among *T*. *cruzi* isolates. Most importantly, and taking into account that Gal*f* residues are not found in mammals, we propose Gp35/50 kDa mucins and/or Gal*f* biosynthesis as appealing and novel targets for the development of *T*. *cruzi* transmission-blocking strategies.

## Introduction

Chagas disease, caused by the protozoan parasite *Trypanosoma cruzi*, is a life-long, debilitating illness of major significance in Latin America, and an emergent threat to global public health [1]. *T*. *cruzi* is a very adaptable organism, able to infect a wide range of mammals and more than 140 species of blood-sucking triatomine bugs that act as transmission vectors in endemic areas. This plasticity likely stems from a complex population structure, made up of multiple strains showing remarkable genetic and phenotypic diversity that were grouped into 6 evolutionary lineages termed TcI to TcVI [2].

Development of *T*. *cruzi* in the insect is a quite complex process that begins when bloodstream trypomastigotes are ingested by triatomines upon blood-feeding on an infected mammal (reviewed in [3]). Parasites able to survive the harsh conditions of the insect crop differentiate into replicative epimastigote forms. These progress on to the triatomine midgut, where they bind to the luminal surface epithelium and/or to the perimicrovillar membranes secreted by the underlying epithelial cells. This attachment is a critical step for effective vector colonization and, accordingly, it involves a multiplicity of parasite surface molecules [4–7]. Upon reaching the insect hindgut, i.e. rectal ampoule and Malpighian tubules, epimastigotes undergo various morphological and biochemical changes that accompany their transformation into metacyclic trypomastigotes (reviewed in [8]). These forms bring the infection into mammals when deposited on the skin or mucosa together with insect excreta during a blood-meal. However, and despite its utmost biological and epidemiological relevance, the mechanisms regulating *T*. *cruzi* metacyclogenesis are poorly understood. Several groups have shown that it may be stimulated by nutritional starvation as well as by different environmental cues such as cAMP, metabolic stressors or pH shifts [8]. Epimastigote adhesion to the highly hydrophobic triatomine rectal cuticle is a pre-requisite for this transformation, and *in vitro* studies using highly defined conditions and artificial hydrophobic surfaces as insect tissue surrogates have shown that improvements in the conditions for parasite attachment to the substrate strictly correlate with an increase in metacyclogenesis [9,10]. Electron microscopy studies revealed a prominent role for *T*. *cruzi* surface molecules, and particularly those localizing to the flagellum tip, during the first steps of substrate attachment. This initial and presumably low-affinity contact is followed by modifications on the parasite body and the formation of stabilizing, desmosome-like structures of unknown composition underneath its plasma membrane [8,11].

The entire outer surface of *T*. *cruzi*, including the parasite body, the flagellum and the flagellar pocket, is covered with glycosylphosphatidylinositol (GPI)-anchored glycoconjugates [12,13]. They include glycoinositolphospholipids (GIPLs) [14], and large families of developmentally regulated glycoproteins, such as mucins, mucin-associated surface proteins (MASPs) and Gp85/*trans*-sialidases [12,15–17]. Mucins expressed by both epimastigotes and metacyclic trypomastigotes (henceforth Gp35/50 kDa mucins) are quite heterogeneous molecules that run on SDS-PAGE as double or triple bands in the relative molecular mass range of ~35–50 kDa depending on the parasite strain [15,18]. The genes coding for their polypeptide backbones, termed *TcSMUG S*, define a family of ~20–50 members bearing just a few, mostly conservative polymorphisms and/or differences in the length of the threonine-rich repeats among them [16,19–22]. *TcSMUG S* genes were also shown to be extremely conserved among *T*. *cruzi* strains [16,19–22]. Upon processing of the *N*-terminal signal peptide and the *C*-terminal GPI-anchoring signal, the average predicted molecular mass for the TcSMUG S polypeptides would be ~7 kDa, with threonine representing up to 50% of the residues [16,20]. *TcSMUG L* genes, on the other hand, code for GPI-anchored polypeptides bearing high sequence similarity to TcSMUG S ones, though they undergo different glycosylation and are restricted to the surface of epimastigote forms [20,21].

A particular feature of the *O*-type oligosaccharide chains from Gp35/50 kDa mucins is that they are α-linked to threonine residues in the TcSMUG S polypeptide core via *N*-acetylglucosamine (GlcNAc) instead of *N*-acetylgalactosamine (GalNAc) as found in mammalian mucins. Depending on the parasite strain, up to 20% of these GlcNAcα residues may remain unsubstituted. Alternatively, they may be elongated, and even branched, with various units of (mostly) β-galactose in different types of linkages, thus leading to a quite complex assortment of oligosaccharides (reviewed in [12,23]). This heterogeneity suggests deficiencies in the ‘streamlining’ of the parasite *O*-glycosylation machinery, which may affect the efficiency of GlcNAc addition to different threonine residues and/or the elongation/termination of individual oligosaccharides. In fact, as many as eight different oligosaccharides have been identified in the Gp35/50 kDa mucins of some parasite strains [12,23]. Concurrent expression of multiple TcSMUG S polypeptides bearing slight polymorphisms may also contribute to increase Gp35/50 kDa mucins’ diversity [16,20,21].

In addition to intra-strain heterogeneities, structural (and hence functional) variations among Gp35/50 kDa mucins from distinct parasite strains have been extensively underscored [12,15,18,23,24]. These differences are mostly attributed to variations in the profile of glycosyltransferases, which indeed constitute a complex family in the *T*. *cruzi* genome [25]. Remarkably, and in addition to β-galactopyranose (βGal*p*) residues, TcI parasite strains display β-galactofuranose (βGal*f*) units in the oligosaccharides of their Gp35/50 kDa mucins [12,23]. The *T*. *cruzi* Tulahuen strain classified as TcVI was also shown to bear βGal*f* units on its Gp35/50 kDa mucins *O*-glycans [26]. However, several Tulahuen-derived clones displayed TcI-like features [27–29], suggesting that the original ‘strain’ may have contained a mixture of parasite genotypes. Importantly, the presence or absence of βGal*f* units in the oligosaccharides of Gp35/50 kDa mucins is more likely explained by inter-strain differences in Gal*f* transferase activities rather than by variations in Gal*f* biosynthesis [30]. In fact, non-TcI strains that do not bear βGal*f* units in the *O*-linked oligosaccharides of Gp35/50 kDa mucins are still able to decorate their GIPL glycan cores with βGal*f* residues [31].

Cumulative evidence point to Gp35/50 kDa mucins as key determinants for *T*. *cruzi* insect-to-mammal host switching. In particular, they were shown to contribute in the recognition, signaling and invasion of mammalian host cells/tissues by metacyclic trypomastigotes [15,24,32–35]. Their linked glycans, particularly upon sialylation of terminal βGal*p* units on the parasite surface, seem critical in all these phenomena. However, little is known about the role(s) played by Gp35/50 kDa mucins during *T*. *cruzi* development within the insect vector. Solely based on their abundance, i.e. up to 10^6−7^ molecules per parasite [15], and high resistance to proteases *in vitro* [18], they were proposed to play a protective role against digestive enzymes in the triatomine crop. In this work, we used epimastigote *ex vivo* binding assays together with different biochemical and genetic approaches to explore the interactions established by these molecules along the triatomine digestive tract.

## Materials and methods

### Insects and parasite stocks

Fifth-instar nymphs of *Triatoma infestans* (Hemiptera: Reduviidae), the most important triatomine vector in the Southern cone countries of South America [2], were obtained from a long-standing colony reared by the Centro de Referencia de Vectores, Dirección de Enfermedades Transmisibles por Vectores–Ministerio de Salud de la Nación (Santa María de Punilla, Córdoba, Argentina), where they were fed on hens weekly. Insects were immediately used for experimental purposes upon arrival to the IIB-INTECh, i.e. ~12–15 days after their last non-infectious blood-meal. *T*. *cruzi* clones used in this study were CL Brener (TcVI), derived from CL strain, isolated from *T*. *infestans* in Brazil and Dm28c (TcI), derived from Dm strain, isolated from *Didelphis marsupialis* in Venezuela [36,37]. Epimastigote forms were grown at 28°C in brain-heart tryptose (BHT) medium supplemented with 10% (v/v) Fetal Calf Serum (FCS), as described [38].

### Parasite transfection

FLAG-tagged versions of *TcSMUG S*, *TcSMUG L* and *TSSA-CL* genes have been described [21,39–41]. All of them were subcloned into the *T*. *cruzi* expression vector pTEX-OMNI [41]. For parasite transfection, exponentially growing epimastigotes (3 x 10^8^) from either CL Brener or Dm28c clone were harvested, washed with phosphate-buffer saline (PBS), transferred to a 0.2 cm gap cuvette (Bio-Rad) with 10 μg of purified DNA and electroporated as described [41,42]. Antibiotic selection (500 μg/mL G418, Gibco Laboratories) was sustained over time once stably transfected populations were obtained.

### Purification of Gp35/50 kDa mucins

Wild-type epimastigote forms (1–3 x 10^9^) were delipidated by chloroform/methanol/water (5:10:4 v/v/v) treatment and then subjected to successive butan-1-ol/water partitions as described [43]. Aliquots of fractions enriched in glycoconjugates were resolved by SDS-PAGE, transferred to nitrocellulose membranes (GE Healthcare), and subjected to a slightly modified version of the periodate-Schiff staining technique. Briefly, blots were incubated in the dark for 1 h in 0.1 M acetic acid containing 10 mM sodium periodate. After extensive washings with PBS, membranes were incubated for 5 min in 15 mM glycerol solution followed by 2 h-incubation in the dark with 5 mM biotin hidrazide (Sigma). Membranes were extensively washed with PBS, blocked with PBS supplemented with 0.1% (v/v) Tween 20 and 5% (w/v) Bovine Serum Albumin (BSA), incubated with HRP-conjugated streptavidin (1:5,000; Sigma) for 1 h and developed using Super Signal West Pico chemiluminescent substrate (Thermo Scientific).

### Flow cytometry and indirect immunofluorescence (IIF) assays

Epimastigote forms (1.5 x 10^6^) were washed, blocked in PBS 10% (v/v) FCS, and incubated with mouse monoclonal antibody (mAb) anti-FLAG (clone M2, Sigma, 1:200 dilution), in an ice-water bath followed by Alexa Fluor-conjugated secondary antibodies (1:500 dilution) (Molecular Probes). After several washes with PBS, parasites were resuspended in 300 μL of PBS containing 4% (w/v) *p*-formaldehyde (PBS 4% PFA), extensively washed with PBS and analyzed using FACS CyFLOW Partec and FloMax software as described [41]. Propidium iodide uptake was evaluated by flow cytometry as described [44]. Epimastigote IIF assays using mAb anti-FLAG, and acquisition and processing of images were done as described [41].

### Western and dot blot

For Western blot analysis, total extracts from 1.5 x 10^7^ parasites were resolved into SDS-PAGE (12.5% gels) and transferred onto nitrocellulose membranes. For dot blot assay, 2 μl of extracts prepared with buffer A (see below) corresponding to 2 x 10^6^ parasites, or appropriate dilutions in PBS, were spotted onto nitrocellulose membranes and let dry for 10 min [45]. Both kinds of blots were blocked with PBS containing 0.1% (v/v) Tween 20 and 1% (w/v) BSA, and reacted with the indicated anti-Gp35/50 kDa mucins’ mAb (all of them were used at 1:5,000 dilution) or antiserum followed by IRDye800CW- or IRDye680LT-conjugated secondary antibodies, and signal intensities were quantified using an Odyssey laser-scanning system (Li-Cor Biosciences). Rabbit polyclonal anti-FLAG antibody (Sigma) and rabbit antiserum to *T*. *cruzi* glutamate dehydrogenase were used as described [21].

### FLAG-affinity chromatography

Pellets of transgenic epimastigotes over-expressing different FLAG-tagged products were resuspended (at 5 x 10^8^ per mL) in ice-cold buffer A (150 mM NaCl, 50 mM Tris.HCl pH 7.6, 1 mM EDTA, 0.1% (v/v) Nonidet P40, 1% (v/v) Triton X-100, and 1 mM PMSF) and incubated on ice for 1 h. After centrifugation, supernatants were incubated with 25 μL of M2 clone mAb anti-FLAG-Sepharose (Sigma), and both the flow-through and retained fractions were evaluated by Western blot.

### Binding of recombinant and native Gp35/50 kDa mucins to insect tissues

Samples resuspended in buffer A as above were normalized by anti-FLAG dot blot assays. Fractions containing similar amounts of ‘FLAG equivalents’ were incubated with sections of the digestive tracts of freshly dissected *T*. *infestans* collected 10 days after a non-infectious blood meal. Following washes with PBS, tissues were fixed with PBS 4% PFA for 30 min, blocked with PBS 10% BSA for 1 h and processed for IIF assay. Briefly, tissue samples were washed and incubated for 1 h with mAb anti-FLAG clone M2 in PBS 5% BSA (1:500 dilution), washed with PBS, and incubated for 1 h with secondary Alexa Fluor-conjugated antibodies in PBS 5% BSA (1:500 dilution). Samples were extensively washed with PBS and mounted with FluorSave Reagent (Sigma). All these procedures were carried out at room temperature. Images were obtained with an IX-81 microscope attached with a FV-1000 confocal module; the objective was a PLAN APO 60X NA 1.42 oil immersion and the acquisition software used was FV 10-ASW 3.1 (all from Olympus Life Sciences, Japan). Images were treated using ImageJ 1.45s Software (NIH, USA) for final presentation. The same procedure was followed to assess binding of native Gp35/50 kDa mucins. In this case, however glycoconjugate-rich samples were partially purified from wild-type epimastigotes by butan-1-ol/water partitions as described above, and IIF assays were developed using mouse mAb 3F5 (at 1:500 dilution) as primary antibody.

### Peptides

Peptides were custom synthesized by GenScript. Their sequences were derived from the predicted *N*-terminal region of mature TcSMUG S (VEAGEGQDQTC, smugS peptide) and TcSMUG L (AVFKAAGGDPKKNTTC, smugL peptide) products, respectively [21]. The *C*-terminal cysteine residue, not present in TcSMUG S/TcSMUG L sequences, was included for eventual peptide coupling purposes. Additional peptides displaying the scrambled sequences of either the smugS peptide (ADEVQDEGQTTT, smugSsc peptide) or the smugL peptide (AAGGVFDKAKPKTTT, smugLsc peptide) were used as controls. Stock solutions (at 10 mg/mL) of all of these peptides were prepared in PBS.

### Carbohydrates

The following carbohydrates were chemically synthesized as indicated and used for the adhesion assays: GlcNAcα-benzyl (Bn) (compound **1**) [46], Gal*f*β1-6GlcNAcα-Bn (compound **2**) [47]; Gal*p*β1-6GlcNAcα-Bn (compound **3**) [48]; Gal*f*β1-4GlcNAcα-Bn (compound **4**) [49]; Gal*f*β1-3GlcNAcα-Bn (compound **5**) [47]; Gal*p*β1-4Glcβ-Bn (compound **6**), Gal*f*β1–4[Gal*p*β1–6]GlcNAcα-Bn (compound **7**) [48]; Gal*p*β1–2[Gal*p*β1–3]Gal*p*β-Bn (compound **8**) [50]; Gal*p*β1-3Gal*p*β1–6[Gal*f*β1–4]GlcNAcα-Bn (compound **9**) [51]; Gal*p*β1–2[Gal*p*β1–3]Gal*p*β1–6[Gal*f*β1–4]GlcNAcα-Bn (compound **10**) [52]; Gal*p*β1–2[Gal*p*β1–3]Gal*p*β1–6[Gal*f*β1-2Gal*f*β1–4]GlcNAcα-Bn (compound **12**) [53], Gal*f*β1-2Gal*f*β1-4GlcNAcα-Bn (compound **13**) [54], Gal*p*β1-2Gal*f*β1-4GlcNAcα-Bn (compound **14**) [54]. The synthesis of pentasaccharide Gal*p*β1-3Gal*p*β1–6[Gal*f*β1-2Gal*f*β1–4]GlcNAcα-Bn (compound **11**) will be described elsewhere. Oligosaccharides were used as Bn glycosides, because they are more stable than the free sugars and we reasoned that the lipophilic benzyl group may improve hydrophobic interactions. All of them were readily solubilized in PBS at 20 mM (stock solutions).

### Epimastigote *ex vivo* adhesion assays

Stationary epimastigotes were suspended in BHT to a density of 10^4^ cells/mL. The content of intermediate forms and/or metacyclics in these parasite suspensions were in the range of 10–16% for CL Brener lines and 15–19% for Dm28c lines. Samples (200 μL) of this parasite suspension together with *T*. *infestans* midguts or rectal ampoules, freshly dissected and washed in PBS, were placed in 96-well microplates and incubated for 30 min at 25°C. When indicated, insect tissues were previously incubated (30 min, 25°C) in PBS supplemented with the indicated peptide (at 0.1 μg/mL [6] unless otherwise stated) or synthetic carbohydrate (at 20 nM [4], unless otherwise indicated). Treated tissues were then washed in fresh PBS and immediately added with 200 μL of the above mentioned parasite suspension. After incubation (30 min, 25°C), insect tissue preparations were spread onto glass slides to expose their inner surface and the number of attached parasites was counted. A Zeiss microscope with reticulated ocular was used for counting parasites attached to 100 randomly chosen epithelial cells in 10 different fields of each tissue preparation. For each experimental group, 10 insects were used and experiments were performed in triplicate. Results were analysed using ANOVA and Tukey's tests using the Graph Pad Prism 6 software.

### Epimastigote *in vivo* infection assays

Even though it is thought that strains of *T*. *cruzi* are able to effectively complete their life cycles, albeit with differential efficiency, in most triatomine vectors [55], to our knowledge, infection of *T*. *infestans* with the Dm28c clone has not been reported. To address this issue, which may have otherwise imposed certain restrictions in our conclusions, we carried out epimastigote *in vivo* infection assays (S1 Fig). Briefly, fifth-instar nymphs of regularly fed *T*. *infestans*, which had been starved for 15 days after the last ecdysis, were fed on artificial bloodmeal apparatus with a mixture of heat-inactivated heparinized rabbit blood and Dm28c culture epimastigotes (1 x 10^9^ parasites in 40 mL of blood) as previously described [6]. At days 14 and 28 post-feeding, the entire digestive tracts consisting of anterior midgut (stomach), posterior midgut and rectum of 20 insects were dissected and homogenized in 500 μL of PBS and the number of infected insects as well as the number of parasites (epimastigotes + metacyclics + intermediate forms)/infected triatomine in each homogenate was determined using a Neubauer hemocytometer.

## Results

### Homologous expression and characterization of recombinant Gp35/50 kDa mucins

**Fig 1:**
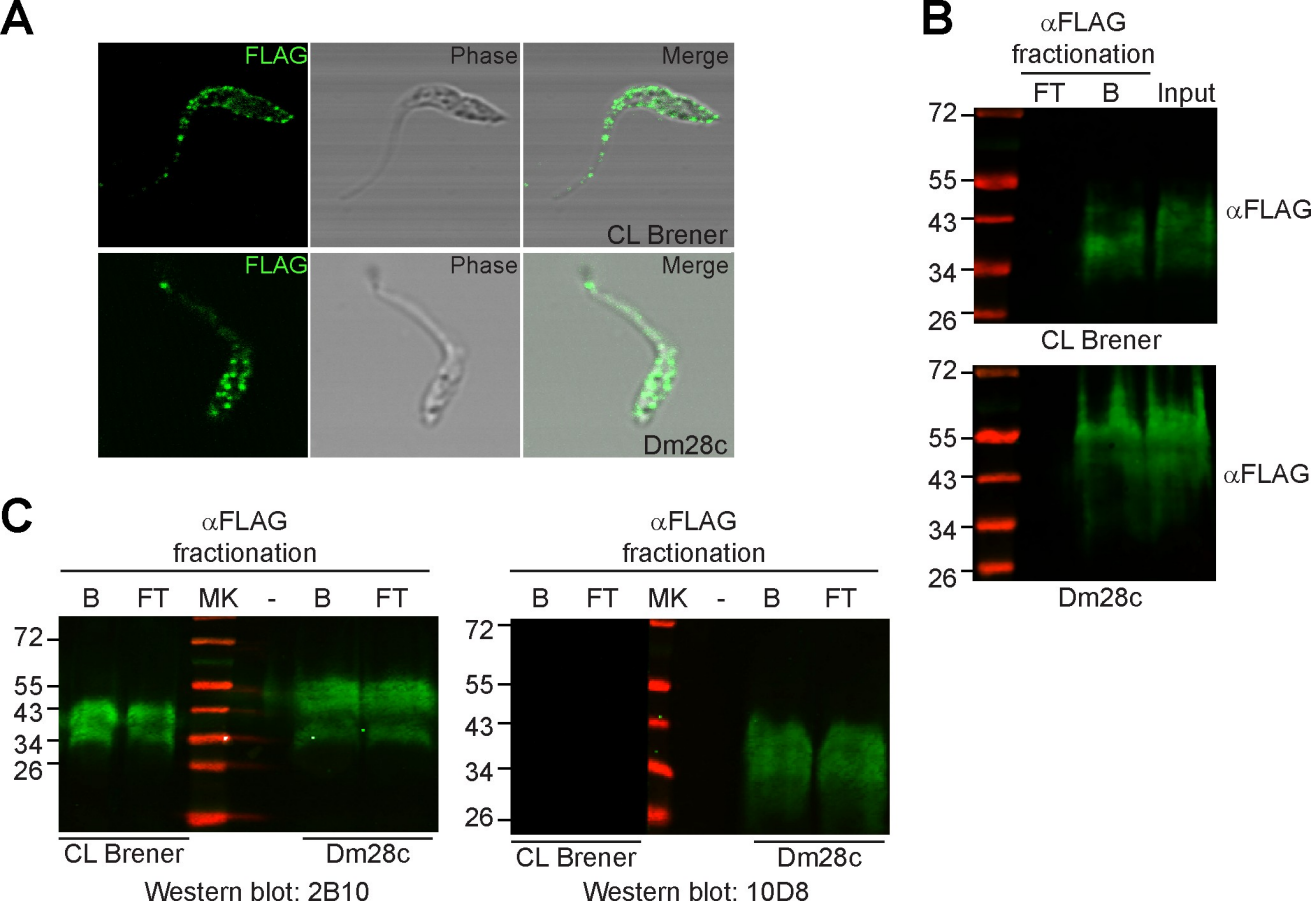
Homologous expression and characterization of recombinant Gp35/50 kDa mucins. **A)** Indirect immunofluorescence assays of permeabilized epimastigotes over-expressing TcSMUG S (TcSMUG S ox) from the indicated parasite clone revealed with mAb anti-FLAG. **B)** Lysates from TcSMUG S ox epimastigotes from the indicated parasite clone were fractionated on to anti-FLAG-Sepharose and input, flow-through (FT) and bound (B) fractions were probed with an anti-FLAG polyclonal antibody. **C)** Aliquots of the FT and B fractions were probed either with mAb 2B10 or mAb 10D8, recognizing Gal*p-* and Gal*f-*based glycotopes restricted to *T*. *cruzi* Gp35/50 kDa mucins, respectively. In **B** and **C**, the positions of relative molecular mass markers (in kDa) are shown.

As a first step to explore the role of Gp35/50 kDa mucins in *T*. *cruzi*-triatomine interplay, we generated transgenic epimastigotes ectopically expressing a FLAG-tagged *TcSMUG S* gene. Transgenic lines (henceforth, TcSMUG S ox lines) were developed into Dm28c and CL Brener, two *T*. *cruzi* clones belonging to extant parasite evolutionary lineages and showing differences on the carbohydrate composition of their Gp35/50 kDa mucins’ *O*-glycans. In particular, Dm28c (TcI) is a ‘Gal*f*-containing’ clone whereas CL Brener (TcVI) is a ‘Gal*f*-lacking’ clone [12,23]. Expression of recombinant Gp35/50 kDa mucins in both genetic backgrounds was initially evaluated by non-permeabilising flow cytometry assays using mAb anti-FLAG. As shown in S2 Fig, FLAG-tagged Gp35/50 kDa mucins were displayed on the surface of both TcSMUG S ox lines, compatible with the proper processing of their predicted sorting signals [42]. IIF assays further supported this surface localization, and revealed that recombinant Gp35/50 kDa mucins are arranged on the membrane of transgenic epimastigotes following a rather patchy distribution (Fig 1A). Clustering and/or aggregation of native mucins (and other surface molecules) on membrane micro-domains has been described in different developmental stages of *T*. *cruzi* [13,21,41,56,57].

To further characterize recombinant Gp35/50 kDa mucins, lysates from TcSMUG S ox parasites were prepared, subjected to anti-FLAG-affinity chromatography, and the different fractions analyzed by Western blot. FLAG-reactive species migrated in reducing SDS-PAGE as a broad smear ranging from 34 to 45 kDa in CL Brener and from 34 to 60 kDa in Dm28c (Fig 1B). Since FLAG-tagged Gp35/50 kDa mucins were expressed upon a unique *TcSMUG S* trans-gene, it can be concluded that intra-clone heterogeneities were due to differences in their post-translational processing, particularly glycosylation. ‘Bound’ and ‘flow-through’ fractions were next probed with mouse mAbs 2B10 and 10D8, which recognize βGal*p-* and βGal*f-*based glycotopes, respectively, on Gp35/50 kDa mucins [18]. Western blots showed that recombinant (enriched in the ‘bound’ fraction) and native (enriched in the ‘flow-through’ fraction) Gp35/50 kDa mucins exhibited rather indistinguishable electrophoretic mobility (Fig 1C). Most importantly, they also revealed that both kinds of glycoprotein display the same pattern of recognition by mAbs directed to Gp35/50 kDa mucins’ glycotopes. Briefly, those expressed by Dm28c epimastigotes bore βGal*f*- and βGal*p*-based glycotopes whereas those expressed by CL Brener parasites exhibited βGal*p*-based structures but lacked βGal*f*-based glycomarkers (Fig 1C). Together, these data indicate that recombinant Gp35/50 kDa mucins expressed by TcSMUG S ox epimastigotes undergo similar post-translational processing and surface display than endogenous molecules and, hence constitute suitable tools for carrying out functional studies.

### Gp35/50 kDa mucins bind to *T*. *infestans* hindgut tissues

**Fig 2:**
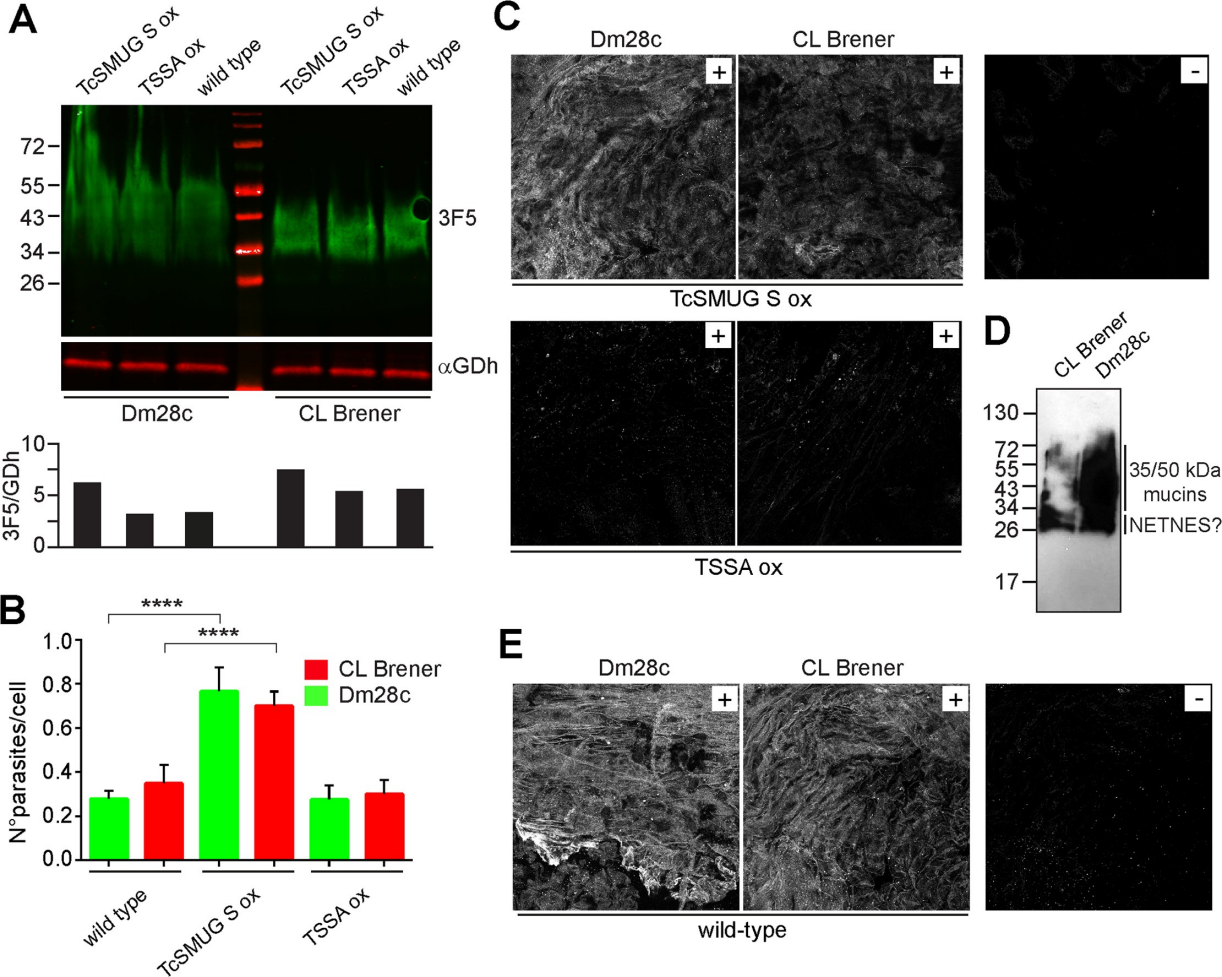
Binding of *T*. *cruzi* Gp35/50 kDa mucins to *T*. *infestans* rectal ampoule. **A)** Total lysates from TcSMUG S ox, TSSA ox and wild-type epimastigotes from either CL Brener or Dm28c clone were analyzed by Western blot using mAb 3F5 and anti-*T*. *cruzi* glutamate dehydrogenase serum (αGDh). The ratio between both signals (3F5/GDh) was calculated by densitometric analyses (lower panel). **B)** Rectal ampoules obtained from *T*. *infestans* fifth-instar nymphs 12–15 days after the last bloodmeal were incubated with 2 x 10^3^ epimastigotes from the indicated line, and the number of adhered parasites per 100 epithelial cells were counted in 10 different fields of each tissue preparation. For each experimental group, 10 insects were used and experiments were performed in triplicate. Data are expressed as mean ± S.D. and asterisks denoted significant differences between the population means (*P <* 0.0001) assessed by ANOVA and Tukey's tests. **C)** Confocal laser microscopy of the inner surface of rectal ampoules of *T*. *infestans* incubated with total lysates from the indicated transgenic line, followed by mAb anti-FLAG-based indirect immunofluorescence (IIF) assay. A control staining of the anti-FLAG mAb in the absence of parasite lysate is shown (-). **D)** Delipidated wild-type epimastigotes from CL Brener or Dm28c clone were extracted with butan-1-ol, and fractions enriched in glycoconjugates were analyzed by SDS-PAGE and periodate-Schiff staining for carbohydrates. The positions of relative molecular mass markers (in kDa) are shown. **E)** Confocal laser microscopy of the inner surface of rectal ampoules of *T*. *infestans* incubated with glycoconjugates from the indicated parasite clone followed by mAb 3F5-based IIF assay. Control staining of mAb 3F5 in the absence of glycoconjugates is shown (-).

To estimate the extent of Gp35/50 kDa mucins over-expression in transgenic epimastigotes, comparative Western blot assays were carried out on CL Brener and Dm28c wild-type and TcSMUG S ox lines. These blots were revealed with mAb 3F5, which recognizes a conserved peptide epitope on Gp35/50 kDa mucins [18], and with an antiserum to *T*. *cruzi* glutamate dehydrogenase (GDh, Fig 2A). The ratio between both signals (3F5/GDh) was calculated by densitometric analyses. As shown in the lower panel of Fig 2, an increase (~25–35% in CL Brener and ~100% in Dm28c) in the overall Gp35/50 kDa mucins’ content of TcSMUG S ox lines as compared to parental lines was recorded. These differences are most likely attributed to FLAG-tagged molecules.

For *ex vivo* binding assays, stationary growing epimastigotes were incubated with different sections of the digestive tract (intestine or rectum) of freshly dissected *T*. *infestans*, and the number of parasites attached per 100 insect cells determined by light microscopy. As shown in Fig 2B, epimastigotes from TcSMUG S ox lines exhibited a significant increase (~2–3 fold) on the *ex vivo* attachment to insect rectal ampoules as compared to wild-type counterparts. To evaluate a possible non-specific effect of transfection and/or growing the parasites under constant drug pressure on this phenotype, we evaluated in parallel the adhesion of parasite lines over-expressing a FLAG-tagged trypomastigote small surface antigen (TSSA) molecule. TSSA is a *T*. *cruzi* glycoprotein restricted to the surface of bloodstream trypomastigote forms [29], involved in adhesion/signaling of non-macrophagic mammalian cells [39,41]. Development of TSSA ox lines in Dm28c and CL Brener genetic backgrounds, as well as the evaluation of surface display of FLAG-tagged TSSA molecules in these transgenic parasites were done as described above (S2 Fig). TSSA ox epimastigotes, expressing roughly equivalent amounts of native Gp35/50 kDa mucins (Fig 2A), displayed similar binding rates to *T*. *infestans* hindgut tissues than parental parasites (Fig 2B). Overall, and even though we cannot formally rule out the possible contribution of additional surface adhesin(s) whose expression/processing may become deregulated in TcSMUG S ox parasites, these data strongly suggest a role for surface-associated Gp35/50 kDa mucins in mediating the interaction between *T*. *cruzi* epimastigotes and the rectum of *T*. *infestans*.

To directly assess the binding of recombinant Gp35/50 kDa mucins to triatomine hindgut tissue, total extracts from TcSMUG S ox and TSSA ox epimastigotes of both genetic backgrounds were prepared and the relative concentration of FLAG-tagged glycoproteins on these lysates calculated by dot blot assays (S2 Fig). Fractions containing similar amounts of ‘FLAG-equivalents’ were then incubated with *T*. *infestans* hindgut tissues and processed for IIF assays using mAb anti-FLAG. As shown in Fig 2C, FLAG-tagged Gp35/50 kDa mucins expressed by either Dm28c or CL Brener transgenic lines displayed significantly improved binding as compared to FLAG-tagged TSSA products, which in turn yielded equivalent signals than those recorded for negative controls. We next assessed the capacity of native Gp35/50 kDa mucins to adhere to triatomine hindgut tissues. To that end, glycoconjugates were purified in bulk from wild-type epimastigotes of Dm28c or CL Brener clone following a standard butan-1-ol extraction protocol. As shown in Fig 2D, periodate-Schiff staining of this material revealed a broad smear ranging from 34 to 60 kDa that most likely corresponded to Gp35/50 kDa mucins and, just below, a duplet of bands that may correspond to NETNES, a small glycoprotein identified and characterized by Macrae et al [58]. Upon incubation of this material with *T*. *infestans* hindgut tissues, direct binding of Gp35/50 kDa mucins was evaluated by IIF assays using mAb 3F5. In line with recombinant Gp35/50 kDa mucins data (Fig 2C), native Gp35/50 kDa mucins from both Dm28c and CL Brener parasite clones interacted with the inner lining of *T*. *infestans* rectal ampoules (Fig 2E). Specificity of the signals was assessed using *T*. *infestans* tissue samples processed for mAb 3F5-based IIF assays in the absence of *T*. *cruzi* purified glycoconjugates (Fig 2E).

### Gp35/50 kDa mucins do not bind to *T*. *infestans* midgut tissues

**Fig 3:**
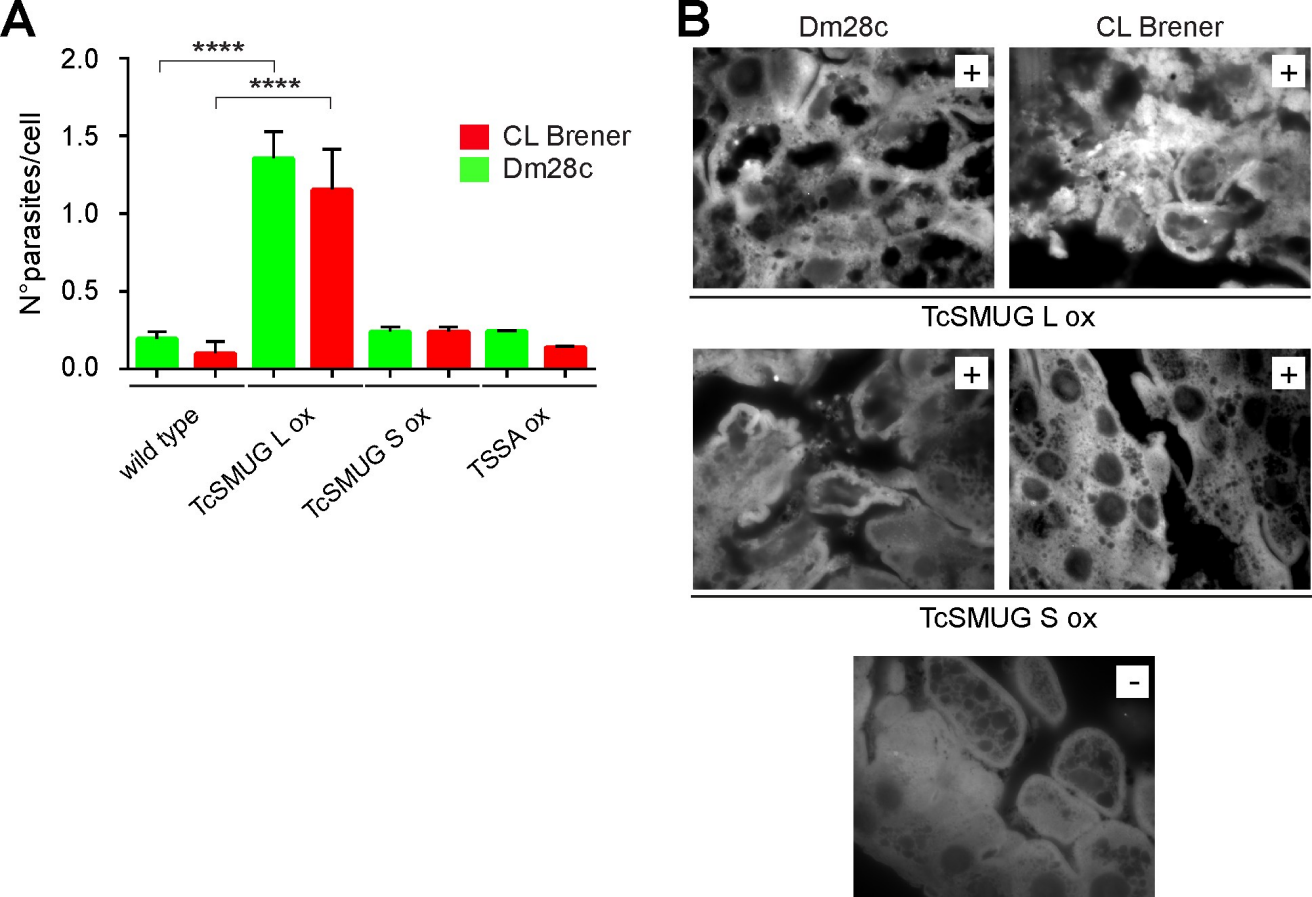
Binding of *T*. *cruzi* TcSMUG L products to *T*. *infestans* midgut tissues. **A)** Midgut tissues obtained from *T*. *infestans* fifth-instar nymphs 12–15 days after the last bloodmeal were incubated with 2 x 10^3^ epimastigotes from the indicated line and the number of adhered epimastigotes per 100 epithelial cells were counted in 10 different fields of each tissue preparation. For each experimental group, 10 insects were used and experiments were performed in triplicate. Data are expressed as mean ± S.D. and asterisks denoted significant differences between the population means (*P <* 0.0001) assessed by ANOVA and Tukey's tests. **B)** Confocal laser microscopy of the inner surface of *T*. *infestans* midguts incubated with total lysates from the indicated transgenic line, followed by mAb anti-FLAG-based indirect immunofluorescence assay. A control staining of the anti-FLAG mAb in the absence of parasite lysate is shown (-).

Similar *ex vivo* binding assays were carried out using freshly dissected triatomine midguts instead of hindgut tissues. In this case, however, surface over-expression of Gp35/50 kDa mucins did not have an effect on the attachment of epimastigotes (Fig 3A). As shown, the number of TcSMUG S ox parasites attached per 100 insect midgut cells was rather indistinguishable than those recorded for TSSA ox lines or wild-type counterparts. As a putative positive control for this experiment, we evaluated the attachment of epimastigotes over-expressing FLAG-tagged TcSMUG L molecules (TcSMUG L ox lines). TcSMUG L are *T*. *cruzi* glycoproteins bearing high sequence similarity to TcSMUG S polypeptides, though they undergo different glycosylation, i.e. they are not acceptors of sialic acid, and are restricted to the surface of epimastigote forms [20,21]. As previously reported by our group [6], a synthetic peptide derived from the conserved *N-*terminal region of TcSMUG L molecules adhered to the luminal endothelium of *Rhodnius prolixus* midguts. Transgenic TcSMUG L ox lines were developed into Dm28c and CL Brener as above, and the expression and surface display of FLAG-tagged TcSMUG L molecules assessed by non-permeabilizing flow cytometry (S2 Fig). When evaluated in *ex vivo* binding assays, TcSMUG L ox lines exhibited significantly increased attachment (~6–8 fold) to *T*. *infestans* midgut tissues as compared to TcSMUG S ox, TSSA ox or wild-type lines (Fig 3A). Unfortunately, differences in the direct binding of recombinant TcSMUG L glycoproteins and recombinant Gp35/50 kDa mucins to *T*. *infestans* midgut preparations could not be properly assessed due to the non-specific reactivity displayed by our developing system (mAb anti-FLAG/secondary antibody to mouse IgG) towards these insect tissues (Fig 3B). Notwithstanding this, and together with published data [6], results of this section support a role of TcSMUG L molecules, but not Gp35/50 kDa mucins, in mediating *T*. *cruzi* parasites anchoring to triatomine midgut tissues.

### Molecular determinants of Gp35/50 kDa mucins’ adhesion to insect hindgut

**Fig 4:**
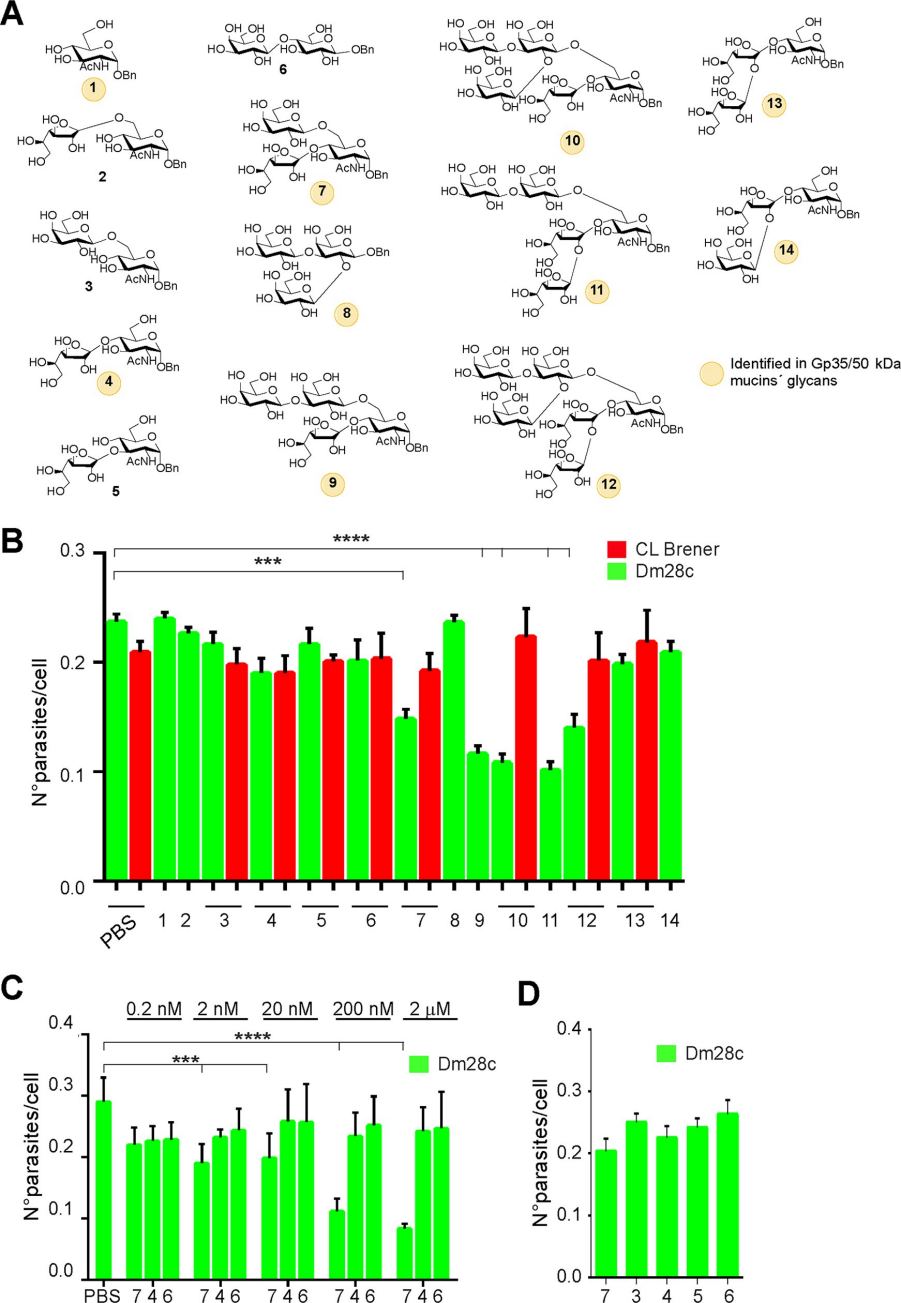
*O*-linked oligosaccharides mediate adhesion of Gp35/50 kDa mucins to *T*. *infestans* rectal ampoule. **A)** Schematic representation of the structures of the oligosaccharides synthesized and assayed in this work. **B)** Hindguts obtained from fifth-instar nymphs 12–15 days after the bloodmeal were incubated for 30 min in PBS or PBS supplemented with the indicated compound (as numbered in A; 20 nM each) and added with interaction medium containing 2 x 10^3^ CL Brener or Dm28c epimastigotes. Adhered epimastigotes were counted per 100 epithelial cells in 10 different fields of each hindgut preparation. For each experimental group, 10 insects were used and experiments were performed in triplicate. **C)** Inhibition assays were carried out as in **B** but using different concentrations of the indicated oligosaccharides. **D)** Inhibition assays were carried out as in **B** but compounds (20 nM each) were added 30 min after the epimastigotes. Data are expressed as mean ± S.D. and asterisks denoted significant differences (*** when *P <* 0.001; and **** when *P <* 0.0001) between each of the indicated populations and the control (PBS treated) population, as assessed by ANOVA and Tukey's tests.

The molecular determinants involved in Gp35/50 kDa mucins’ adhesion to *T*. *infestans* rectal ampoule were next explored by carrying out *ex vivo* epimastigote binding assays in the presence of compounds that may act as competitors. To that end, a panel of oligosaccharides were chemically synthesized as described in Materials and Methods. They include different mono- to hexasaccharides found in the *O*-linked glycans of Gp35/50 kDa mucins obtained as α-benzyl glycosides (compounds **1**, **4**, **7**, and **9**–**14**); the trisaccharide **8,** which is part of oligosaccharides **10** and **12,** as β-benzyl glycoside; and appropriate controls (Fig 4A). *T*. *infestans* hindgut tissues were individually pre-incubated with these carbohydrates (at 20 nM) for 30 min, washed with PBS and then used to perform epimastigote *ex vivo* binding assays as before. As shown in Fig 4B, αGlcNAc-Bn, equivalent to the common reducing end of Gp35/50 kDa mucins’ *O*-linked oligosaccharides, did not interfere with epimastigote binding. Neither did disaccharides containing αGlcNAc in different linkages to βGal*p* or βGal*f* units (compounds **2**–**5**), nor the lactose derivative (compound **6**), used as control (Fig 4B). In contrast, the branched trisaccharide Gal*f*β1–4[Gal*p*β1–6]GlcNAcα-Bn (compound **7**), yielded significant inhibition (Fig 4B). Different linear and branched trisaccharides, some of which were also identified in the glycans of Gp35/50 kDa mucins (compounds **8**, **13** and **14**) presented negligible effect (Fig 4B). These results indicated a specific inhibitory effect for compound **7** and, together with the fact that this compound bears the ‘negative’ Gal*f*β1-4GlcNAcα (compound **4**) and Gal*p*β1-6GlcNAcα (compound **3**) motifs, suggested that both its constituent carbohydrates and its branched structure contribute to its inhibitory effect.

Most notably, compound **7** was able to exert an inhibitory effect on the binding of Dm28c but not on CL Brener epimastigotes to the insect rectal ampoule (Fig 4B), strongly suggesting that it is competing with similar structures, i.e. Gal*f*β1–4[Gal*p*β1–6]GlcNAcα motifs, present in Dm28c Gp35/50 kDa mucins’ glycans but absent in CL Brener ones. In line with this, additional assays showed that branched tetrasaccharide (compound **9**), pentasaccharides (compounds **10** and **11**), and hexasaccharide (compound **12**), all of them bearing the Gal*f*β1–4[Gal*p*β1–6]GlcNAcα motif, also interfered with the interaction between Dm28c, but not CL Brener, epimastigotes and *T*. *infestans* hindgut tissues (Fig 4B). Though not significantly different, compounds **9**–**12** showed a slight increase in their inhibitory capacity as compared to compound **7** (Fig 4B). The inhibitory effect of compound **7** could not be attributed to non-specific effects on parasite motility and/or viability (S3 Fig) and was dose-dependent (Fig 4C), further supporting that its mode of inhibition is by blocking a potential ligand-receptor interaction involved in Dm28c epimastigote-*T*. *infestans* rectal ampoule recognition. Interestingly, if this compound was added after incubation of the parasites with the hindgut tissue, its inhibitory effect was not observed (Fig 4D).

**Fig 5:**
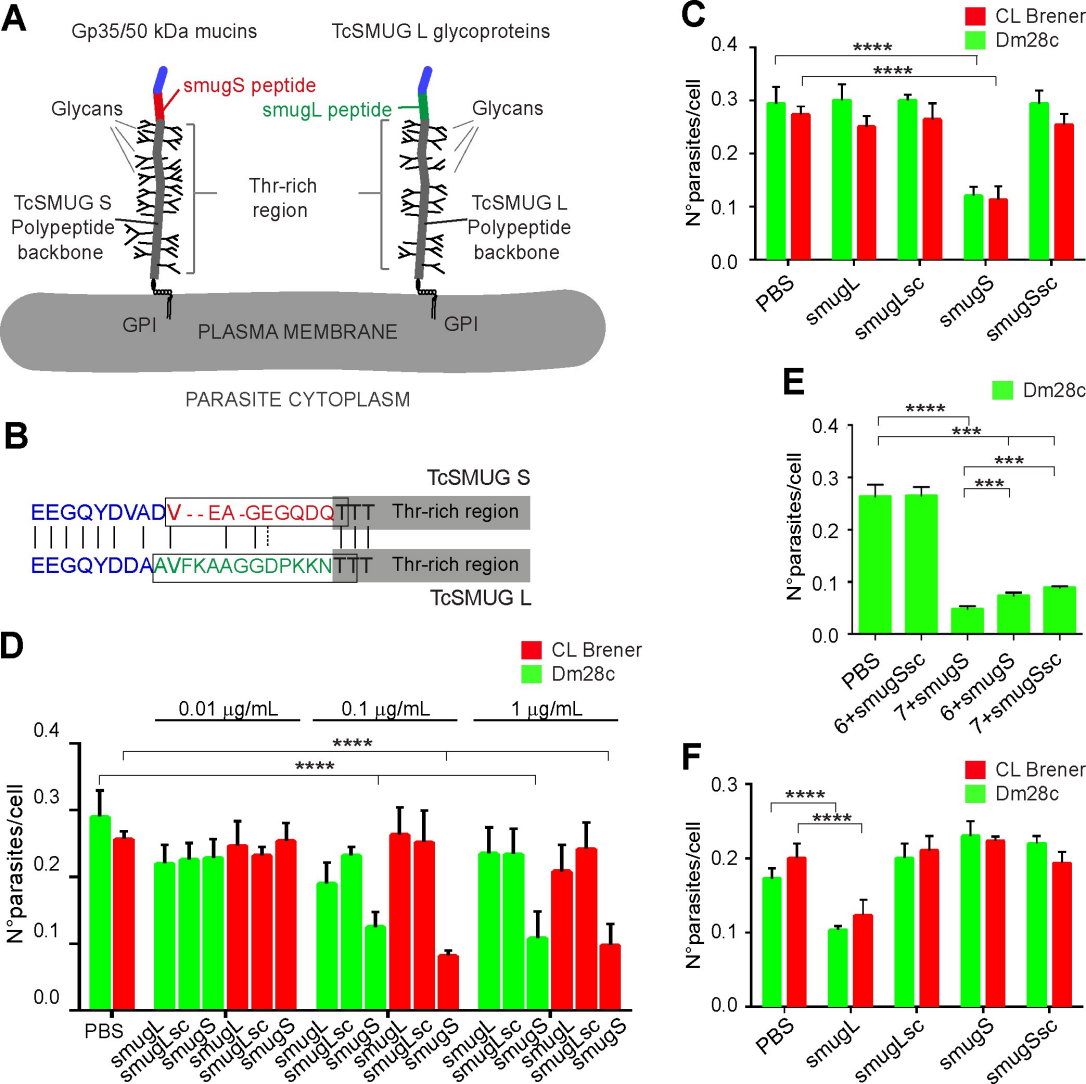
Peptidic determinants mediate adhesion of Gp35/50 kDa mucins to *T*. *infestans* rectal ampoule. **A)** Schematic diagram showing the structural features and topological disposition of surface-displayed Gp35/50 kDa mucins and TcSMUG L glycoproteins. The relative position of the smugS and smugL peptides on the overall polypeptide is indicated. GPI, glycosylphosphatidyl inositol. **B)** Sequence alignment between the mature *N-*terminal regions from TcSMUG S and TcSMUG L canonical proteins. Amino acids are colorized in accordance to their relative position on the polypeptide, as defined in **A**. Residues included in the smugS and smugL peptides are boxed. **C-F)** Freshly dissected rectal ampoules **(C-E)** or midgut tissues **(F)** obtained from fifth-instar nymphs 12–15 days after the last bloodmeal were incubated for 30 min in PBS or PBS supplemented with the indicated synthetic peptide (at variable concentrations in panel **D**; at 0.1 μg/mL in panels **C** and **F** and added with interaction medium containing 2 x 10^3^ CL Brener or Dm28c epimastigotes. In panel **E**, tissues were incubated for 30 min in PBS or PBS supplemented with the indicated synthetic peptide (0.1 μg/mL) and carbohydrate compound (as numbered in Fig 4A; 20 nM each). In all cases, adhered epimastigotes were counted per 100 epithelial cells in 10 different fields of each hindgut preparation. For each experimental group, 10 insects were used and experiments were performed in triplicate. Data are expressed as mean ± S.D. and asterisks denoted significant differences (*** when *P <* 0.001; and **** when *P <* 0.0001) between each of the indicated populations and the control (PBS treated) population, as assessed by ANOVA and Tukey's tests. In panel **E**, additional pairwise comparisons were carried out (compound **7** + smugS peptide vs compound **6** + smugS peptide and compound **7** + smugS peptide vs compound **7** + smugSsc peptide), and results are indicated as above.

The fact that CL Brener Gp35/50 kDa mucins lack βGal*f*-based glycotopes and thereby Gal*f*β1–4[Gal*p*β1–6]GlcNAcα-bearing structures on their *O*-linked glycans [12,23], suggested the existence of additional molecular determinant(s) underlying their adhesion to *T*. *infestans* hindgut tissues (Fig 2). To address this issue, we tested the inhibitory potential of a synthetic peptide (smugS peptide) from the mature *N*-terminal region of TcSMUG S proteins on *ex vivo* epimastigote binding assays. Topological reconstructions indicate that this region, which cannot undergo glycosylation, protrudes from the sugar-coated structure of Gp35/50 kDa mucins (schematized in Fig 5A) [20,21]. As controls, we used a peptide spanning the ‘corresponding’ *N*-terminal sequence of TcSMUG L glycoproteins (smugL peptide) and peptides spanning a scrambled version of either sequence (smugSsc and smugLsc peptide) (Fig 5A and 5B). As shown in Fig 5C and 5D, attachment of epimastigotes to *T*. *infestans* rectal ampoule could be partially counteracted (in a dose-dependent manner) by preincubation with the smugS peptide, but not with PBS or control peptides. At variance with the compound **7** (Fig 4B), the smugS peptide displayed a similar inhibitory effect on the binding of both CL Brener and Dm28c epimastigotes (Fig 5C and 5D), which correlates with its high degree of conservation among TcSMUG S deduced polypeptides from different parasite strains [20,21]. Most interestingly, the inhibitory effect of a mixture containing both the smugS peptide and compound **7** was significantly higher than those recorded for the individual reagents (Fig 5E), suggesting they are interacting with different molecular partners on triatomine hindgut tissues. As shown for compound **7**, the inhibitory capacity of the smugS peptide could not be attributed to non-specific effects on parasite motility and/or viability (S3 Fig).

To further assess the specificity of the latter results, we also tested the effect of synthetic peptides on epimastigote-*T*. *infestans* midgut interaction. In this case, and in line with transgenic parasite data (Fig 3) and previous results [6], the smugL peptide but neither the smugS nor the scrambled peptides significantly interfered with epimastigote adhesion (Fig 5F). Overall, these data indicate that the smugS peptide from the mature *N*-terminal region of the TcSMUG S scaffold polypeptide and the Gal*f*β1–4[Gal*p*β1–6]GlcNAcα motif from the *O*-linked glycans are molecular determinants of Gp35/50 kDa mucins’ adhesion to the triatomine rectal ampoule.

## Discussion

The *T*. *cruzi* taxon comprises multiple strains showing remarkable genetic and phenotypic diversity [2]. In susceptible triatomine models, this diversity translates into large variations in the rate of parasite proliferation and/or transmissibility [59–62]. Although underexplored, the cellular basis underlying such differences are thought to be related to the dissimilar capacity of parasite strains to withstand the action of haemolytic factors, antimicrobial peptides and/or the insect gut microbiota [61,63–68]. Alternatively, or additionally, these variations may be related to the dissimilar profile of interactions established by different parasite strains with receptor(s) along the digestive tract of triatomines. In line with this framework, we herein show that natural variations in the *O*-glycosylation of Gp35/50 kDa mucins modulate *T*. *cruzi* epimastigote anchoring to the triatomine rectal ampoule, which may in turn lead to differences in parasite differentiation/transmissibility to the mammalian host.

At variance with all of the epimastigote surface receptors characterized so far [4–7], including the TcSMUG L glycoproteins analyzed in this work, Gp35/50 kDa mucins display ‘tropism’ towards triatomine hindgut rather than midgut tissues. Experimental evidence supporting this conclusion could be summarized as follows: i) native and recombinant Gp35/50 kDa mucins directly bind to the internal cuticle of *T*. *infestans* rectal ampoules; ii) transgenic epimastigotes over-expressing Gp35/50 kDa mucins on their surface coat display an exacerbated *ex vivo* attachment to such tissues; and iii) chemically synthesized compounds derived from Gp35/50 kDa mucins are able to specifically interfere with epimastigote attachment to *T*. *infestans* rectal ampoule, most likely by competing with or directly blocking epimastigote surface-insect receptor(s) pairing. By means of competition assays, two different adhesion determinants were identified in Gp35/50 kDa mucins: the smugS peptide, derived from the mature *N*-terminus of TcSMUG S protein scaffolds, and the Gal*f*β1–4[Gal*p*β1–6]GlcNAcα motif, derived from their *O*-linked glycans. Both compounds display an additive inhibitory effect, strongly suggesting that they engage different receptors on the inner lining of *T*. *infestans* hindgut tissues. Interestingly, the Gal*f*β1–4[Gal*p*β1–6]GlcNAcα motif impaired the attachment of Dm28c (TcI) but not of CL Brener (TcVI) epimastigotes to triatomine rectal ampoules; which is in line with the distribution of Gp35/50 kDa mucins bearing this trisaccharide across the *T*. *cruzi* taxon [12,23].

Topological reconstructions suggest that the smugS peptide is ideally suited for the engagement of counter-receptors on insect tissues. Upon being tethered to the outer layer of the parasite membrane by a *C*-terminal GPI motif and undergoing extensive glycosylation in the secretory pathway, the threonine-rich region of surface Gp35/50 kDa mucins is predicted to adopt a rigid, ‘stalk-like’ structure. This architecture, in turn, projects the outermost and non-glycosylated *N*-terminal region of the TcSMUG S polypeptides above the parasite glycocalix. Variations on this theme, aimed at improving the exposition of peptidic ligand-binding domains of surface molecules by means of heavily glycosylated regions have been proposed for TcSMUG L glycoproteins [6] as well as for other *T*. *cruzi* surface molecules [16,69] and a multiplicity of yeast flocculins and adhesins [70].

βGal*f* residues have been shown to play a role in maintaining membrane/cell wall physiology and/or in enhancing the virulence of *Leishmania* and certain pathogenic bacteria and fungi [71,72]. Our current findings suggest that Gal*f* residues are also major forces driving the interaction between *T*. *cruzi* surface glycoconjugates and the digestive tract of triatomine vectors. In line with this, it is worth mentioning that the involvement of Gal*f* residues on the *T*. *cruzi*-triatomine interplay has been previously proposed by Nogueira *et al* [4]. Briefly, these authors have shown that biochemically purified GIPLs from epimastigotes of the Y strain (TcII) adhere to *R*. *prolixus* midgut tissues. Treatment of such GIPLs with diluted trifluoroacetic acid partially abolished their binding capacity, thereby suggesting a role of terminal non-reducing Gal*f* residues decorating GIPLs’ glycan on triatomine midgut recognition. Whether *T*. *cruzi* GIPLs are also able to interact with triatomine hindgut tissues is being currently explored. However, and even in the case they do, the contrasting structures of the Gal*f*β1–4[Gal*p*β1–6]GlcNAcα motif and the GIPLs’ glycan core (in which Gal*f* residues are linked to α-mannopyranose units instead of α-GlcNAc residues [14]), along with the fact that Gp35/50 kDa mucins do not bind to *T*. *infestans* midgut endothelium argue against the possibility that GIPLs and Gp35/50 kDa mucins may be using the same triatomine molecular partner(s). The synthetic molecules and transgenic parasites developed here, along with recently released triatomine genomes [73,74], may provide helpful tools to identify the interacting partner(s) of Gp35/50 kDa mucins (and eventually of *T*. *cruzi* GIPLs) on the inner lining of the triatomine rectal ampoule.

Overall, the most parsimonious hypothesis to explain our results would imply that the smugS peptide and Gal*f*β1–4[Gal*p*β1–6]GlcNAcα motifs on Gp35/50 kDa mucins engage with different insect counter-receptors during the initial steps of *T*. *cruzi* epimastigote binding to *T*. *infestans* hindgut tissues. In the latter case, such counter-receptor(s) is/are expected to display lectin-like properties. The occurrence of digestive lectins recognizing carbohydrate-based motifs has been demonstrated in other vector-borne protozoa and viruses [75–77]. Moreover, a pioneer work, not further pursued, described a series of putative lectins with diverse monosaccharide specificities in different regions of the digestive tract of *R*. *prolixu*s [78]. The sheer number of Gp35/50 kDa molecules on the epimastigote surface [15], together with the clustering effect achieved by means of their organization on membrane micro-domains [13,56] and/or by the presence of several oligosaccharides carrying the Gal*f*β1–4[Gal*p*β1–6]GlcNAcα motif in a single TcSMUG S polypeptide may increase the overall affinity/avidity of these interactions, as shown in other models [79,80]. Supporting this idea, our data show that epimastigote adhesion, mediated by ‘aggregating’ surface adhesion determinants, cannot be disrupted if the ‘non-aggregating’ Gal*f*β1–4[Gal*p*β1–6]GlcNAcα-Bn compound is added after incubation of the parasites with the triatomine hindgut tissue. Other epimastigote surface molecules, including GIPLs and/or NETNES (although no Gal residues were found linked to this molecule [58]) may also contribute to initial parasite-hindgut interaction, which should be later on strengthened by the formation of desmosome-like structures of unknown composition underneath the epimastigote plasma membrane [8,11]. Upon metacyclogenesis, parasites readily detach from the triatomine rectal ampoule cuticle, a process that is supposed to occur because of changes on the composition of their surface coat [8,81]. Differentiation to metacyclic forms also correlates with an up-regulation of surface *trans*-sialidase activity, and with a massive sialylation of terminal βGal*p* units in Gp35/50 kDa mucins’ oligosaccharides [12]. In this framework, a regulatory effect of sialic acid incorporation on the adhesion properties of Gal*f*β1–4[Gal*p*β1–6]GlcNAcα-containing glycans might be proposed.

Despite continuous efforts, the prospects for the development of effective vaccines and/or appropriate drugs for large-scale public-health interventions against Chagas disease are still clouded by substantial scientific and socioeconomic challenges [82]. In this scenario, development of novel drugs, novel drug targets and/or novel strategies to control parasite transmission are urgently needed. Based upon our findings, we propose Gp35/50 kDa mucins and/or Gal*f* biosynthesis as appealing targets for intervention. Since adhesion to the insect hindgut strictly correlates with epimastigote differentiation into infective forms, the development of compounds able to interfere with this interaction and their subsequent delivery into triatomines by transgenic and/or paratransgenic technologies [83–85] is expected to have an impact into *T*. *cruzi* vector transmissibility and, hence in Chagas disease epidemiology.

## Supporting information

S1 Fig Susceptibility of *T. infestans* to infection with the *T. cruzi* Dm28c clone. Insects (*n* = 40) were fed on heparinized, complement-inactivated rabbit blood containing Dm28c epimastigotes. Insects were dissected at days 14 (*n* = 20) or 28 (*n* = 20) post-feeding and the number of infected insects (expressed as %) is shown in panel **A.** The number of total flagellates (including epimastigotes, metacyclics and intermediate forms) in the whole gut of infected insects were determined by microscopy and expressed as mean ± S.D. in panel **B**.
(TIF)

S2 Fig Expression and relative quantitation of FLAG-tagged products on transgenic *T. cruzi* epimastigotes. **A)** Non-permeabilized CL Brener or Dm28c epimastigotes were labeled with mAb anti-FLAG and evaluated by flow cytometry. Parasites over-expressing TcSMUG S (TcSMUG S ox), TSSA-CL (TSSA ox) or TcSMUG L (TcSMUG L ox) are shown in pink, green and blue, respectively. Wild-type parasites are indicated in light blue and isotype labeling control is depicted in red. **B)** Lysates of epimastigotes from the indicated transgenic line were diluted as indicated in PBS, spotted in triplicate on nitrocellulose membranes and assayed by mAb anti-FLAG-based dot-blot revealed using IrDye800CW-conjugated anti-mouse antibody. **C)** Densitometric analyses of signals shown in **B**.
(TIF)

S3 Fig Viability of epimastigotes after oligosaccharide/peptide incubation. Viability of epimastigotes from the indicated lines after treatment with the indicated peptide or carbohydrate compound (see Fig 4A for numbering) was assessed by propidium iodide uptake and analyzed by flow cytometry. The genetic background of the parasite line (CL, CL Brener; Dm, Dm28c) is indicated.
(TIF)
