# Supplementary figures and images for: Trypanosoma cruzi surface mucins are involved in the attachment to the Triatoma infestans rectal ampoule
Source: PLoS Negl Trop Dis. 2019 May 20;13(5):e0007418. doi: 10.1371/journal.pntd.0007418 (PMC6544316; doi:10.1371/journal.pntd.0007418)

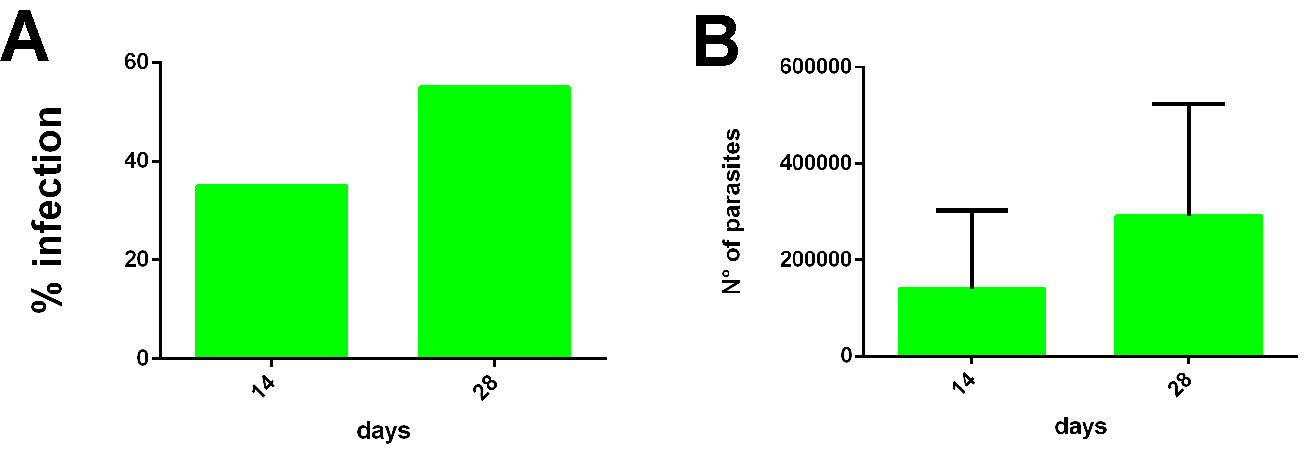

Supplement: S1 Fig — Insects (n = 40) were fed on heparinized, complement-inactivated rabbit blood containing Dm28c epimastigotes. Insects were dissected at days 14 (n = 20) or 28 (n = 20) post-feeding and the number of infected insects (expressed as %) is shown in panel A. The number of total flagellates (including epimastigotes, metacyclics and intermediate forms) in the whole gut of infected insects were determined by microscopy and expressed as mean ± S.D. in panel B. (TIF) [file pntd.0007418.s001.tif]

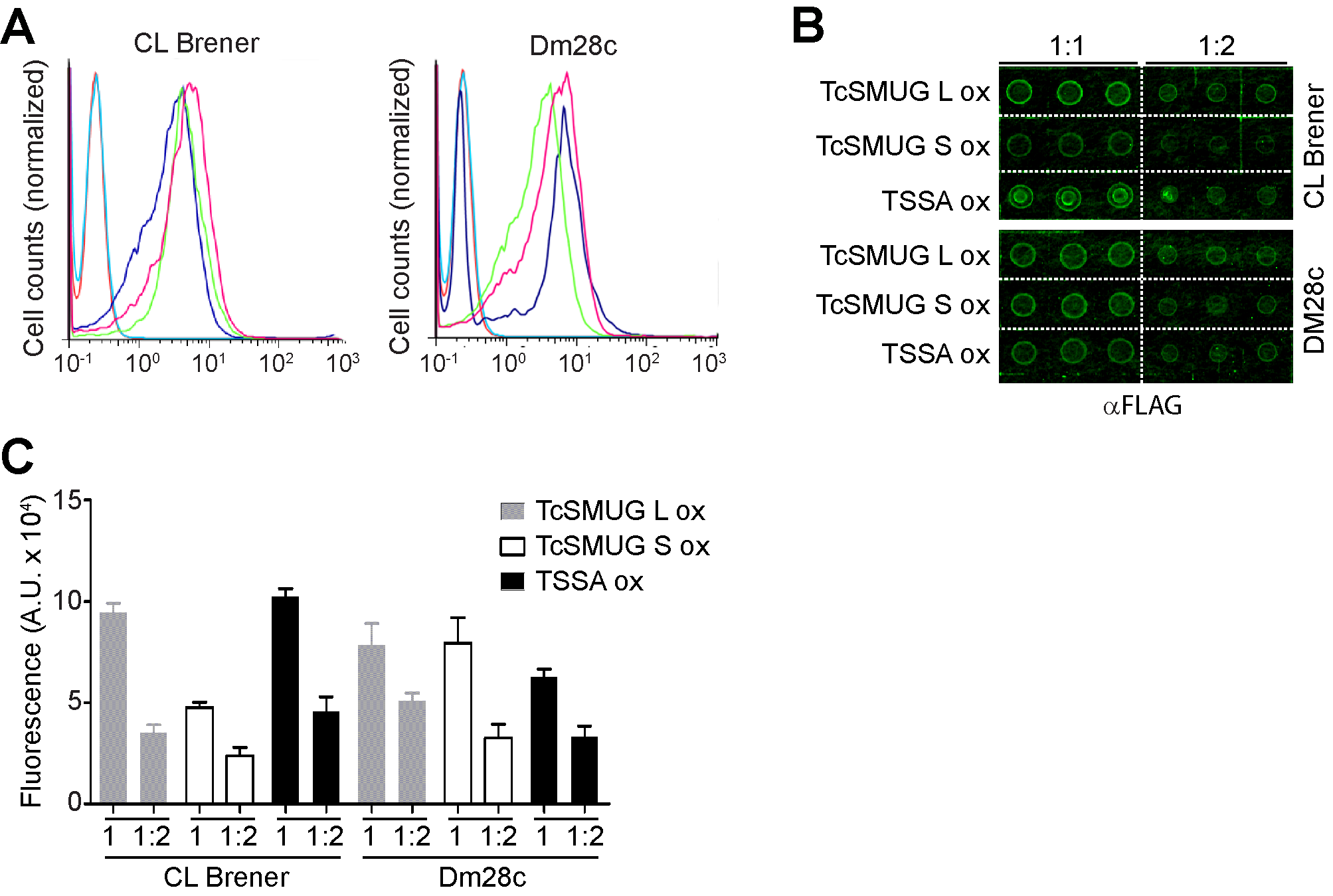

Supplement: S2 Fig — A) Non-permeabilized CL Brener or Dm28c epimastigotes were labeled with mAb anti-FLAG and evaluated by flow cytometry. Parasites over-expressing TcSMUG S (TcSMUG S ox), TSSA-CL (TSSA ox) or TcSMUG L (TcSMUG L ox) are shown in pink, green and blue, respectively. Wild-type parasites are indicated in light blue and isotype labeling control is depicted in red. B) Lysates of epimastigotes from the indicated transgenic line were diluted as indicated in PBS, spotted in triplicate on nitrocellulose membranes and assayed by mAb anti-FLAG-based dot-blot revealed using IrDye800CW-conjugated anti-mouse antibody. C) Densitometric analyses of signals shown in B. (TIF) [file pntd.0007418.s002.tif]

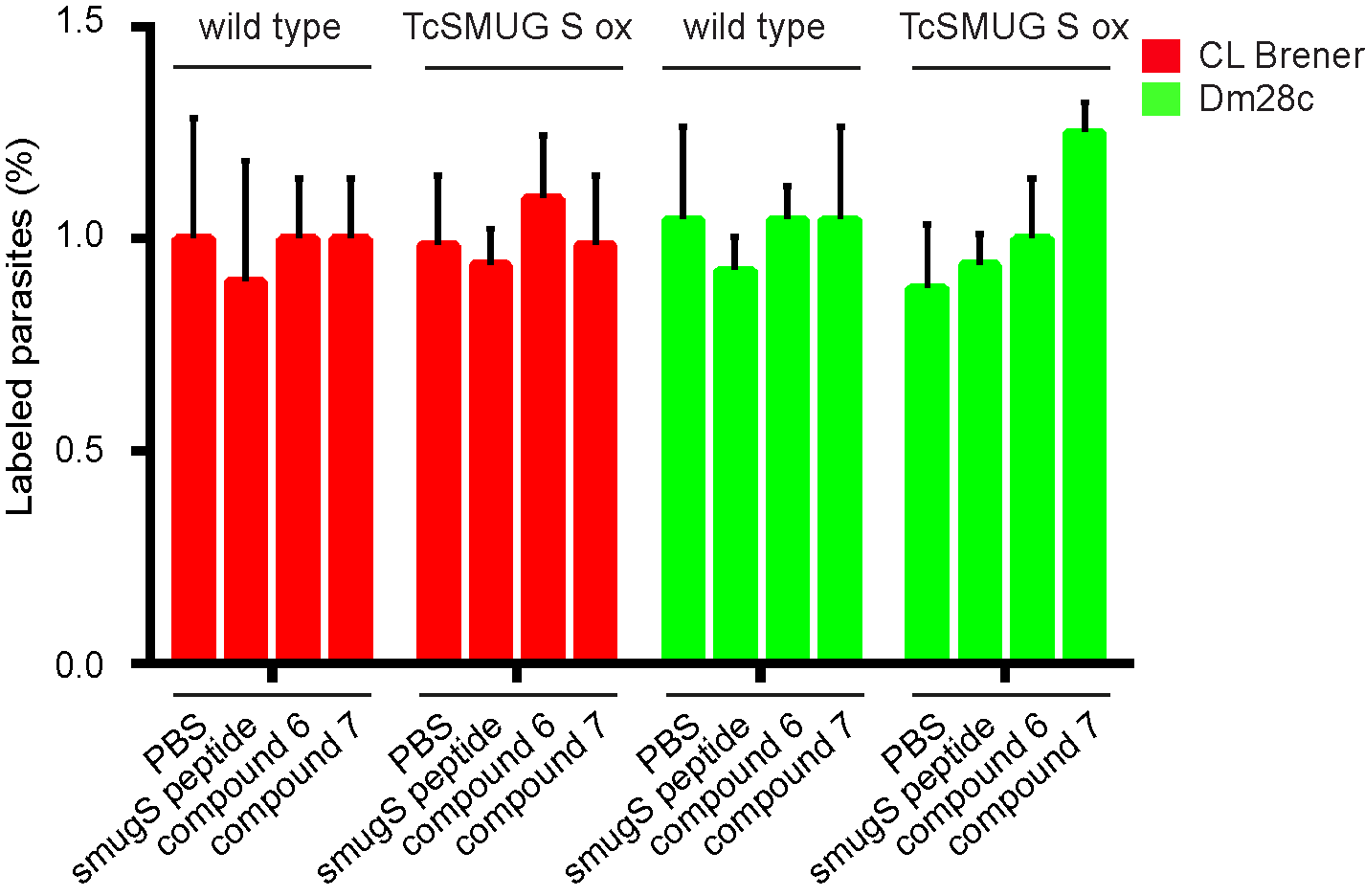

Supplement: S3 Fig — Viability of epimastigotes from the indicated lines after treatment with the indicated peptide or carbohydrate compound (see Fig 4A for numbering) was assessed by propidium iodide uptake and analyzed by flow cytometry. The genetic background of the parasite line (CL, CL Brener; Dm, Dm28c) is indicated. (TIF) [file pntd.0007418.s003.tif]
